# Patterns of Facial Profile Preference in a Large Sample of Dental Students: A Cross-Sectional Study

**DOI:** 10.3390/ijerph18168554

**Published:** 2021-08-13

**Authors:** Lívia Romsics, Angyalka Segatto, Kristóf Boa, Roland Becsei, Noémi Rózsa, László Párkányi, Ildikó Pinke, József Piffkó, Emil Segatto

**Affiliations:** 1 Department of Oral and Maxillofacial Surgery, Faculty of Medicine, University of Szeged, H-6725 Szeged, Hungary; livy092@gmail.com (L.R.); boa.kristof@gmail.com (K.B.); becsei.roli@gmail.com (R.B.); piffkojozsef@gmail.com (J.P.); 2 Segatto Dent’art Studio, H-1015 Budapest, Hungary; ng.segatto@gmail.com; 3 Department of Paediatric Dentistry and Orthodontics, Faculty of Dentistry, Semmelweis University, H-1085 Budapest, Hungary; rozsa.noemi@dent.semmelweis-univ.hu; 4 Department of Periodontology, Faculty of Dentistry, University of Szeged, H-6720 Szeged, Hungary; parkanyilaci@gmail.com; 5 Department of Orthodontics and Pediatric Dentistry, Faculty of Dentistry, University of Szeged, H-6720 Szeged, Hungary; pinkeildiko@gmail.com

**Keywords:** dental education, facial esthetics, facial profile

## Abstract

The objective of this study was to explore dental students’ facial profile preferences in a large sample of students. Nine hundred and nineteen dental students of four dental schools were involved. As part of a larger study on dentofacial esthetics, six photo series consisting of one unaltered and four altered variants of the same female profile were distributed among the students. The altered features were ones that are esthetically significant according to the literature. The students had to indicate the photo in each series that they preferred. The data were analyzed in a regression model in which preference in the given photo series was the dependent variable and gender, grade of studies, and dental school were the factors. Eight hundred and sixty-one students (93.7%) responded. Gender and dental school were not associated with the observed preferences, but the grade of studies was associated for three of the modified parameters: chin prominence, the sagittal position of the maxillary dental arch, and the simultaneous modification of the prominence of the chin and the nose. This study has confirmed several earlier observations, and new observations have also been made. We have demonstrated that the anteroposterior position of the maxillary incisors may be an important determinant of profile esthetics, even if this position does not influence the situation of the soft tissues and if the forehead cannot be used as a reference. We have also shown that the harmony between the nose and the chin overrides the importance of their individual dimensions.

## 1. Introduction

Since the 1980s, several studies have investigated whether and how dental education (the process of becoming a dental professional) influences one’s perception of dental and facial esthetics. Some studies have discussed the outcome, that is the difference between dental professionals and laypersons in this respect [1,2,3], while others have concentrated directly on the process of dental education [4,5]. Research in this area is important in two ways. First, any potential mismatch between the dentist’s and patient’s perceptions and expectations in this respect carries the risk of a result that is esthetically unacceptable for the patient. Second, identifying areas of profession-specific esthetic perception that are significantly influenced by dental education can inform dental curricula. Nowadays, there is agreement in the literature that dental professionals and laypersons do differ in their perception of certain aspects of dentofacial esthetics.

Studies in dentofacial esthetics approach their subject predominantly in two aspects: part of the studies discuss “frontal” aspects, such as the esthetics of the teeth, the smile, or the harmony of the face [4,5,6,7], while others deal with the “lateral” aspect, that is the facial profile [3,8,9]. Naturally, the results of such studies usually show high geographical/cultural variety, which indicates that they are best used to identify critical parameters or critical areas of perception (i.e., ones that potentially vary to a great degree across laypersons and dental professionals) rather than to find an “esthetic universal”. Such factors and areas, then, can be consciously used in either treatment planning or education.

The esthetic perception of facial profile is less often and less deeply studied among dental students than the above-mentioned frontal esthetic aspects. Furthermore, when it is studied in this context, it happens most often in connection with class II/III malocclusion and its correction [3,10,11,12]. While malocclusion due to the malposition of the mandible is indeed an important clinical problem with a bearing on profile esthetics [13], other—often connected and interacting—factors are also at play: the length of the nose [14], the prominence of the soft tissues of the chin [15], the ratio of the nose to the lips [16], or the inclination of the maxillary incisors [17] have all been reported to influence the perception of profile esthetics by both laypersons and dental professionals.

Between October 2018 and August 2019, our research group conducted a large, cross-sectional multi-site study on the dentofacial mini- and microesthetic preferences of dental students. The study involved 861 students from all five grades of all four of the Hungarian dental faculties [18]. In addition to the main instrument, we distributed six photo series of modified facial profiles among the respondents and asked them to indicate the image they preferred best in each series. Our aim was to determine if the preferences were associated with the respondents’ gender, the grade of dental school they attended, or the institution they attended. As this additional assessment was only loosely related to the focus of the main study, we decided to publish the results separately. In the present study, we discuss these results.

## 2. Materials and Methods

### 2.1. Subjects and Sampling

All five grades of all four of the dental faculties of Hungary (associated with the universities of Szeged, Debrecen, Pécs, and Budapest) were involved in the study. Printed questionnaires were distributed among the faculties to study the dentofacial mini- and microesthetic preferences of dental students. For details about the development of the questionnaire, please see our previous publication [18]. The album with the profile photo series for rating (see below) was included as a supplement to these questionnaires. Altogether, 1011 questionnaires were distributed to 919 Hungarian-speaking students of the four institutions. The authors were present in person when the questionnaires were administered to ensure adherence to the standard instructions and procedures. Thirty minutes were allocated for answering the questions (including the photo rating). Sampling took place between October 2018 and August 2019.

The study protocol and the applied instruments (including the profile photo album) were approved by the Regional Ethics Committee for Research in Human Medical Biology at the University of Szeged (No. 178/2018-SZTE). Participation was anonymous and voluntary.

### 2.2. Photo Series

The profile photos for rating were prepared by us. The model was a Caucasian female with normal cephalometric parameters (for Hungary) and without any clinical abnormality that could possibly affect the facial profile. The image was a standard lateral view in portrait orientation showing the lower and middle facial thirds, including the nose, the anterior teeth, the lips, and the chin. The upper edge bordered on the lower rim of the orbit. The photos were taken at a 1.5 m distance from the model, with a Nikon D7000 camera (Nikon Corporatio, Tokyo, Japan) equipped with a Nikon 105 mm F 2.8G VR AF-S ED.IF Nikkor objective (Nikon Corporatio, Tokyo, Japan). The model was standing with a natural head position while being photographed. The photo shooting session took place on a sunny day, in a room amply and evenly lit by natural light, at noontime.

**Figure 1:**
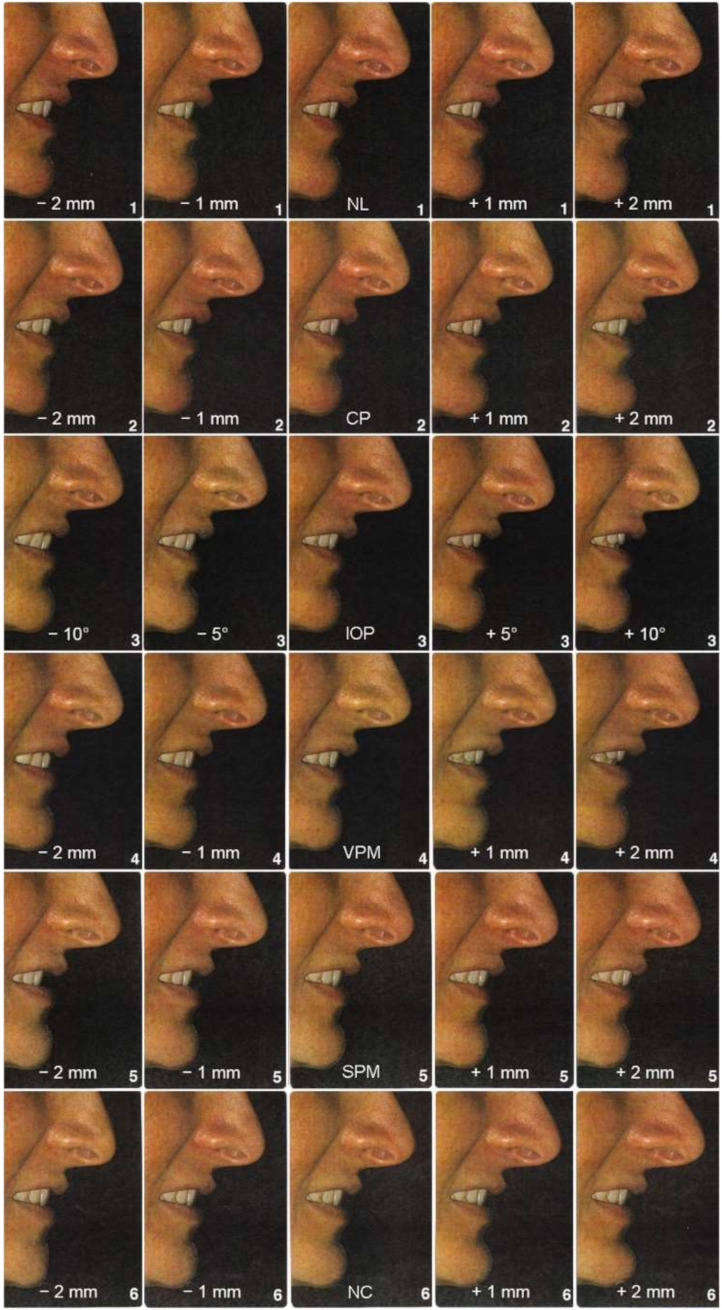
The six series of modified photos. In each series, the unmodified variant is always the middle one. Please note that the photos were not administered to the respondents in this format, but instead were in a photo album, in random order, and without any labeling. This collage was created only as an illustration to ease the interpretation of the results. The numbers in the bottom right corner of the individual images (1 to 6) denote the number of the series. NL: nose length, CP: chin prominence, IOP: occlusal plane inclination, VPM: vertical position of maxillary arch, SPM: sagittal position of the maxillary arch, NC: nose length and chin prominence.

Six photo series were used. For all six series, the original (unmodified) photo and four modified versions of the photo were used, so all series (defined by the modified feature or features) consisted of five photos (Figure 1). Modifications were always made to the original image (i.e., no modification was generated by further modification of a previously modified version.) For the modifications, Adobe Photoshop CC 2015 (Adobe Systems, San Jose, CA, USA) was used. The 30 photos were arranged in an album in a way that items from the same series were always shown on the same page, in random order (not as shown in Figure 1, where the photos are arranged to ease the interpretation of the results). The respondents were not told which photo was the unmodified version. The modified features were ones that have been reported in the literature to influence facial esthetics. The modifications are summarized in Table 1.

### 2.3. Statistical Analysis

Statistical analysis was carried out by an independent evaluator (see the Acknowledgements section). The analysis was blind: the evaluator received coded results and was told what analyses to carry out using what coding, without knowledge of the meaning of the codes.

The results were analyzed in SPSS 22.0 (IBM Corp., Armonk, NY, USA). Continuous variables were characterized with means and standard deviations or medians. Categorical variables were described with the number of observed cases and frequencies expressed in percentages. To test the contribution of students’ gender, grade, and institution to the variability of their preferences, a multinomial regression model was built, in which students’ preferences were entered as the dependent variable, and the said predictors were entered as factors. The reference grade was always Grade 1, and the reference image was always the unmodified image. This analysis was performed for each of the six photo series. The null hypothesis was that none of the factors had significant contribution to the variability of student preferences. The level of significance was *p* = 0.05, unless otherwise indicated.

## 3. Results

### 3.1. Subjects

Of the 919 students, 861 (93.7%) responded. Thus, the initial student pool for the photo rating consisted of 861 students. The rating of the profile photos was accomplished by 843 to 855 students, depending on the given photo series (97.9–99.3%). The mean age of the students in the entire sample was 22.85 (±2.49) years. The sample consisted of 560 females (65%) and 301 males (35%), indicating the known feminization tendency within the dental profession in Hungary. The distribution of students across academic institutions was as follows: 296 students (34.4%) attended Semmelweis University (Budapest, Hungary), 206 students (23.9%) attended the University of Debrecen (Debrecen, Hungary), 218 students attended the University of Szeged (Szeged, Hungary) (25.3%), and 141 students (16.4%) attended the University of Pécs (Pécs, Hungary). These ratios correspond to the relative sizes of the dental schools. The demographic characteristics of the sample broken down by grade are given in Table 2.

### 3.2. Photo Rating

For gender and institution, the null hypothesis was retained for all photo series. The regression analysis indicated a significant effect of grade for photo series 2 (chin prominence, CP, *p* < 0.05), 5 (sagittal position of the maxillary arch, SPM, *p* < 0.05), and 6 (simultaneous modification of nose and chin, NC, *p* < 0.01).

Table 3 shows the preferences for the individual photos in the CP series by grade. According to the results of the regression analysis, students in the fifth (final) grade opted significantly more frequently for the −1 mm modification than the original as compared to the preferences of the first-grade students (odds ratio (OR): 1.998, confidence interval (CI) 95%: 1.078–3.705, *p* < 0.05). In fact, as the table shows, students in the first grade preferred the original image over the −1 mm modification. In the fifth grade, just the opposite was observed.

Preference for a retruded chin was a general tendency: the majority of the choices (>60%) were distributed across the original and the retruded modifications in all grades (approximately evenly, apart from grades 1 and 5). Preference for the original was the lowest in the fifth grade (19.1%) and the highest in the third grade (27.2%), where it was also the most popular choice.

Table 4 summarizes the preferences for the SPM series. Here, a quite peculiar tendency was observed: the popularity of the +2 mm modification constantly rose from the first through the fifth grade (29% to 41.5%). This was also reflected in the result of the regression analysis: fifth graders opted significantly more frequently for the +2 mm modification than the original as compared to the preferences of the first graders (odd ratio (OR): 1.721, confidence interval (CI) 95%: 0.887–3.338, *p* < 0.05). This gain did not happen at the cost of any single modification. Another minor observation was that second graders opted for the −1 mm modification significantly less frequently than first graders (OR: 0.422, CI 95%: 0.212–0.839, *p* < 0.05).

Preference for the original image was the lowest in the fifth grade (12.0%) and the highest in the second grade (27.2%). In none of the grades was the unmodified image the most popular choice. The overall tendency was an increasing preference for a frontally shifted maxillary arch.

Table 5 gives an overview of the preferences for the NC series. The regression analysis indicated that both fourth graders (odd ratio (OR): 0.422, confidence interval (CI) 95%: 0.212–0.839, *p* < 0.05) and fifth graders (odd ratio (OR): 0.397, confidence interval (CI) 95%: 0.208–0.757, *p* < 0.01) opted significantly less frequently for the +1 mm modification than the original as compared to the preferences of the first-grade students. Furthermore, fifth-grade students also opted significantly less frequently for the +2 mm modification (odd ratio (OR): 0.418, confidence interval (CI) 95%: 0.219–0.798, *p* < 0.01). As for the pattern of preferences: the first grade preferred the two extremes (±2 mm), slightly more than half of the respondents opted for either of these. By the second grade, +2 mm and −1 mm dominated the choices. In grades 3 and 4, the −1 mm modification was the most popular, and in the fifth grade, the original, unmodified image was the most frequently chosen. That is, students’ choices appear to have moved from the extremes toward the original from the first through the fifth grades.

As for the rest of the photo series, no significant effect of any of the predictors could be found, and the choices were remarkably evenly distributed across the image versions.

## 4. Discussion

This study sought to determine in a large sample of dental students if the students’ facial profile preferences were associated with their gender, the grade of dental school they attended, or the institution they attended. As the instrument (the photo series) was administered as a supplement to a larger questionnaire on dentofacial esthetics, only these few background variables could be used, which necessarily resulted in poor explanatory power. However, the large sample size still allows for the identification of patterns, and we have confined our analysis to this aspect. Gender and school were not associated with the observed preferences, but the grade of studies was associated for three of the modified parameters.

The lack of gender effect came as no real surprise, as earlier we made the same observation regarding dentofacial esthetics in the same sample [18]. This is most probably a sample-specific feature. It is known that facial esthetic preferences show high geographical (cultural) variability. For instance, Strajnic and colleagues failed to find gender difference in self-perception and satisfaction with dental appearance in a sample of Serbian patients [19], while Przylipiak and co-workers, working in Poland, found that preferred mean nose size was statistically significantly lower in females in comparison with males [16]. The present study also tested nose size preferences but failed to find significant gender differences. It must be added that most of the data in the literature (including the above cited studies) come from samples of laypersons, which makes one wonder if it is dental education that somehow homogenizes an otherwise gender-dependent preference. To answer this goes far beyond the limits of this discussion, but we propose that this hypothesis could generate an intriguing line of research.

As for the lack of significant difference between the individual dental schools, this is a result that we, in fact, expected. Hungary is a small, culturally homogeneous country, which means that students come from virtually the same background in this respect. The dental profession is a small community in which most professionals, including the faculty of the dental schools, know each other or at least each other’s work. This has an immense homogenizing effect on both the material covered and the teaching methodologies. Finally, all dental schools are predominantly state financed. These factors together result in a largely undifferentiated dental education, so the findings in this respect are not surprising at all.

Before we turn to the profile-specific findings, it must be emphasized that in this study, white Caucasian students of both sexes and of a homogeneous cultural background rated images of a white Caucasian female; when we talk about facial esthetics or preferred facial features in connection with the results, it is to be understood within these limitations.

The observed preference patterns suggest that progress in one’s dental studies does influence one’s preferred facial profile features. In this specific sample, this was observed regarding the degree of chin prominence, the sagittal position of the maxillary dental arch, and chin prominence and nose length in combination (i.e., the harmony between the chin and the nose).

A less prominent/slightly retruded chin is a feature generally associated with the female face [20,21]. It is well documented in the literature that observers of various ethnic and cultural backgrounds consider the degree of chin protrusion as a determining feature of facial esthetics. Torsello and colleagues demonstrated in connection with female profiles that, both among laypersons and orthodontists, nose protrusion was more tolerated than a similar amount of chin protrusion [15]. Hongyu and co-workers found that laypersons but not orthodontists put emphasis on chin protrusion when making esthetic judgements about female and male faces [22]. Studying young adult Korean women with a preferred facial appearance (winners of the Miss Korea contest), Kim and colleagues concluded that a retruded chin is a crucial part of what makes a female face perceived as esthetic [23]. Finally, in his study of the esthetic plastic surgical alteration of the chin, Lee recommends that the chin projection should lie ∼3 mm posterior to a line drawn in the nose-lip-chin plane (i.e., it should be slightly retruded) [24]. It seems, thus, that a slightly retruded chin is generally considered to be a key component of an esthetic female face, but the cited studies show that sometimes this applies to male faces too. The results of our study support that point.

We also found that an anteriorly shifted maxillary arch gained popularity toward the higher grades. The most probable explanation is that this is the effect of the position of the maxillary incisors, which is documented to have a significant effect on the esthetics of the facial profile [25]. Most studies explain this effect by the varying degrees of soft tissue displacement [26,27]. This, however, cannot really explain our findings, as the modification of the antero-posterior position of the arch was too small to cause readily perceivable soft tissue changes in real life, especially while smiling—so the soft tissues were not modified in our images. What generated this peculiar pattern of preferences if not soft tissue changes? Andrews [28] found that for a Caucasian female profile to be found harmonious, the maxillary incisors need to be positioned anterior to the forehead’s facial axis (FFA) point and posterior to glabella. The importance of the maxillary incisor–forehead relation has been shown since then in other populations too [29]. Unfortunately, our images were cropped at the lower rim of the orbit, so it was impossible for our respondents to observe these relations. Our results corroborate those of other studies in terms of the importance of the anteroposterior position of the maxillary incisors, but they also show that this feature can have a significant influence on esthetic judgements even if neither soft tissue changes nor the forehead can be used as a reference. It also seems that dental students gradually develop a preference for an anteriorly shifted maxillary arch during their studies. These appear to be hitherto undocumented aspects of facial profile esthetics, which we cannot satisfactorily explain. A study involving laypersons, dentists, and orthodontists would be desirable to learn more about this pattern.

To interpret the results regarding the simultaneous modification of the nose and the chin, let us first see what was observed when these areas were modified separately. As for the chin, we have earlier established that a retruded chin was preferred by most of the respondents. As for the length of the nose, we found no association with any of the background variables (i.e., the preferences were homogeneous in the entire sample). This is apparently in contradiction with studies that found that a shorter nose is perceived as more esthetic than a longer one [14,16]. However, once again, these studies analyzed the preferences of laypersons, so their results are not entirely comparable to ours. Unfortunately, the few available studies that dealt with the facial profile preferences of dental students [3,30] did not discuss the role of the nose, so it is difficult to tell if a relative indifference to the dimensions of the nose is a general characteristic of dental students or just a sample-specific finding. However, what happens when the modifications to the nose and chin are linked? If the prominence of the chin is the dominant feature of the two (as the results suggest), one would expect that the shorter modifications would also dominate in this case. This is not what our results show. As we noted before, students’ choices appear to have moved from the extremes toward the original from the first through the fifth grades. This should not be interpreted in an over-simplifying manner, such as by saying that by the fifth grade of dental school students had seen enough to be able to safely judge what is normal. A glance at the distribution of choices in the fifth grade (Table 5) tell us just the opposite: the distribution is stunningly homogeneous, and the unmodified variant stands out by mere 8%. In our opinion, this shows that (a) the chin and the nose are linked in terms of facial profile esthetics, and (b) any modification to either the prominence of the chin or the nose may be perceived as esthetic if it is accompanied by the same degree of modification to the other. To put it simply, the harmony between the nose and the chin overrides the importance of their individual dimensions. The practical consequence of this observation is that any intervention that potentially modifies the prominence of the nose or the chin should take both structures into consideration.

Finally, the studied factors did not have a significant effect on the preferences for three parameters. Of these, we have already discussed nose length. As for the inclination of the occlusal plane and the vertical position of the maxillary arch, these parameters are usually regarded as important determinants of smile esthetics [31,32,33]. Unfortunately, no studies are available on how they influence the esthetic perception of the facial profile. Our results suggest no significant effect, but we do not wish to draw conclusions from a single observation, even if it has been made in a large sample.

## 5. Conclusions

To our knowledge, no previous study has examined the perception of facial profile esthetics in a similarly large sample of dental students. This study has confirmed several earlier observations made in smaller samples and also resulted in new observations. We have demonstrated that the anteroposterior position of the maxillary incisors may be an important determinant of profile esthetics, even if this position does not influence the situation of the soft tissues and if the forehead cannot be used as a reference. We have also shown that the harmony between the nose and the chin overrides the importance of their individual dimensions. Focused studies are needed to learn if the same patterns may be found in other populations.

## Figures and Tables

**Table 1:** Photo rating: the modified profile features.

Series No.	Item No.	Feature	Description
1	4.1	Nose length (NL)	The length of the nose changes between −2 mm and +2 mm as compared to the original image in 1 mm steps.
2	4.2	Chin prominence * (CP)	The prominence of the chin changes between −2 mm retrusion and +2 mm protrusion as compared to the original image in 1 mm steps.
3	4.3	Occlusal plane inclination (IOP)	The inclination of the occlusal plane changes between 10° downward and 10° upward as compared to the original image in 5° steps. ^#^
4	4.4	Vertical position of maxillary arch (VPM)	The maxillary arch is shifted vertically between −2 mm and +2 mm (downward and upward) as compared to the original image in 1 mm steps.
5	4.5	Sagittal position of the maxillary arch (SPM)	The maxillary arch is shifted sagittally between −2 mm and +2 mm as compared to the original image in 1 mm steps.
6	4.6	Nose length and chin prominence (NC)	The length of the nose and the prominence of the chin change simultaneously between −2 mm and +2 mm as compared to the original image in 1 mm steps.

* Chin prominence was defined as the prominence of the soft tissues of the chin. The position of the mandible was not changed. ^#^ Inclination is represented by the angle.

**Table 2:** Demographics by grade for the entire sample (*N* = 861).

Grade	*N*	Gender (*N* (%))	Age (Mean ± *SD*)
First	187	M: 71 (38%)	19.99 ± 4.03 years
		F: 116 (62%)	
Second	184	M: 61 (33%)	21.40 ± 2.96 years
		F: 123 (67%)	
Third	137	M: 41 (30%)	22.23 ± 5.31 years
		F: 96 (70%)	
Fourth	169	M: 62 (37%)	23.92 ± 3.33 years
		F: 107 (63%)	
Fifth	184	M: 66 (36%)	24.31 ± 3.52 years
		F: 118 (64%)	

M: male, F: female.

**Table 3:** Distribution of preferences across grades regarding chin prominence (CP, photo series No. 2).

			−2 mm	−1 mm	Original	+1 mm	+2 mm	Total
Grade	1	Count	50	34	45	36	21	186
		% within grade	26.9%	18.3%	24.2%	19.4%	11.3%	100.0%
	2	Count	49	39	45	33	17	183
		% within grade	26.8%	21.3%	24.6%	18.0%	9.3%	100.0%
	3	Count	34	34	37	17	14	136
		% within grade	25.0%	25.0%	27.2%	12.5%	10.3%	100.0%
	4	Count	47	37	43	11	28	166
		% within grade	28.3%	22.3%	25.9%	6.6%	16.9%	100.0%
	5	Count	54	53	35	22	19	183
		% within grade	29.5%	29.0%	19.1%	12.0%	10.4%	100.0%
Total		Count	234	197	205	119	99	854
		% within grade	27.4%	23.1%	24.0%	13.9%	11.6%	100.0%

**Table 4:** Distribution of preferences across grades regarding the sagittal position of the maxillary arch (SPM, photo series No. 5).

			−2 mm	−1 mm	Original	+1 mm	+2 mm	Total
Grade	1	Count	19	45	27	41	54	186
		% within grade	10.2%	24.2%	14.5%	22.0%	29.0%	100.0%
	2	Count	16	27	38	50	53	184
		% within grade	8.7%	14.7%	20.7%	27.2%	28.8%	100.0%
	3	Count	20	27	22	24	43	136
		% within grade	14.7%	19.9%	16.2%	17.6%	31.6%	100.0%
	4	Count	20	28	24	28	66	166
		% within grade	12.0%	16.9%	14.5%	16.9%	39.8%	100.0%
	5	Count	18	26	22	41	76	183
		% within grade	9.8%	14.2%	12.0%	22.4%	41.5%	100.0%
Total		Count	93	153	133	184	292	855
		% within grade	10.9%	17.9%	15.6%	21.5%	34.2%	100.0%

**Table 5:** Distribution of preferences across grades regarding the simultaneous modifications of the nose and chin (NC, photo series No. 6).

			−2 mm	−1 mm	Original	+1 mm	+2 mm	Total
Grade	1	Count	35	30	27	48	46	186
		% within Grade	18.8%	16.1%	14.5%	25.8%	24.7%	100.0%
	2	Count	34	46	30	28	46	184
		% within Grade	18.5%	25.0%	16.3%	15.2%	25.0%	100.0%
	3	Count	19	44	23	31	19	136
		% within Grade	14.0%	32.4%	16.9%	22.8%	14.0%	100.0%
	4	Count	33	50	30	25	28	166
		% within Grade	19.9%	30.1%	18.1%	15.1%	16.9%	100.0%
	5	Count	33	34	48	34	34	183
		% within Grade	18.0%	18.6%	26.2%	18.6%	18.6%	100.0%
Total		Count	154	204	158	166	173	855
		% within Grade	18.0%	23.9%	18.5%	19.4%	20.2%	100.0%

## Data Availability

Additional data may be available upon request from the corresponding author.
